# Do English healthcare settings use ‘Choice Architecture’ principles in promoting healthy lifestyles for people with psoriasis? An observational study

**DOI:** 10.1186/s12913-015-0808-1

**Published:** 2015-06-02

**Authors:** Chris Keyworth, Pauline A Nelson, Christopher EM Griffiths, Lis Cordingley, Chris Bundy, on behalf of the Identification and Management of Psoriasis Associated Co-morbidity (IMPACT) Team

**Affiliations:** Centre for Dermatology Research, University of Manchester, Manchester Academic Health Science Centre, Manchester, UK; Dermatology Centre, Salford Royal Hospital, Manchester Academic Health Science Centre, Manchester, UK; Manchester Centre for Health Psychology. University of Manchester, Manchester Academic Health Science Centre, Manchester, UK

**Keywords:** Health promotion, Choice architecture, Behavior change, Nudge, Psoriasis

## Abstract

**Background:**

The influence of environmental factors in shaping behaviour is becoming increasingly prominent in public health policy, but whether health promotion strategies use this knowledge is unknown. Health promotion is important in the management of psoriasis, a long-term inflammatory skin condition, and health centre waiting areas are ideal places to promote health information to such patients. We systematically examined patient information materials containing either general, or specific, health messages for patients with psoriasis.

**Methods:**

An observation schedule was used to record the frequency and quality of leaflets and posters addressing lifestyle behaviour change in health centre waiting areas. Content analysis was used to analyse: frequency, characteristics and standard of the materials.

**Results:**

Across 24 health centres 262 sources of lifestyle information were recorded (median per site = 10; range = 0–40). These were mainly: generic posters/displays of lifestyle support (*n* = 113); and generic materials in waiting areas (*n* = 98). Information quality was poor and poorly displayed, with no high quality psoriasis-specific patient materials evident.

**Conclusions:**

There is little attempt to promote healthy lifestyle as an important aspect of psoriasis management in the clinic environment. Evidence about using environmental cues/techniques to prompt behaviour change in people with psoriasis does not currently inform the design and display of such information in standard health centre settings, which are prime locations for communicating messages about healthy lifestyle. Future research should test the efficacy and impact of theory-informed, high quality health promotion messages on health outcomes for patients with psoriasis.

## Background

Evidence for the role of environmental factors in shaping healthy lifestyle is increasing [1, 2]. A growing body of research is examining the influence of the environment on behavioural choices related to health promotion. Changing the environment in subtle ways can influence a range of behaviours including encouraging healthier food choices and promoting physical activity such as increasing the accessibility to stairs rather than elevators [3–8]. This is referred to as ‘nudge theory’ or ‘choice architecture’ [9].

Whilst the environment can have a detrimental effect on health, such as promoting the availability of ‘fast foods’ and ‘convenience foods’ [10], a number of studies also emphasise its potential in prompting positive behaviour change [6, 7] in a range of settings such as schools, food stores and health care [6]. Something as simple as a change in poster size can positively affect stair use [11].

According to some theorists human behaviour is often automatic, where positive behavioural change is ‘cued’ by environmental stimuli. These often go unnoticed by the individual and therefore absent of any conscious reflection and are considered habitual or unconscious desires [11, 12]. Brief, and somewhat subtle interventions employing this strategy, have proved successful in prompting behavioural change through changing implicit environmental cues [7, 13]. Although evidence that locating visual prompts in a person’s physical environment can serve as cues to action, it is unclear from the current literature if and how health promotion signposting within health care settings (specifically primary and secondary care health centres) is drawing upon the growing evidence related to choice architecture [9].

As part of traditional ‘health promotion’ strategies at the individual level, health care settings have long been recognised as places where patients are exposed to written and visual information about healthy lifestyle. Patient health information leaflets are viewed as a vehicle to improve attitudes towards, and increase knowledge about health and illness behaviour, and promote behaviour change. This information can be used to ‘prompt’ behaviour change. Whilst this strategy may rely on conscious engagement with the information, this is recognised as an intervention strategy, whereby micro-environments can be altered by adding visual prompts [13]. These can be processed by individuals either automatically, or at a more engaged, reflective level [13]. However, for this to happen often complex health messages should be clear, visible and accessible to have the desired impact on behaviour.

Hartley [14] and Houts *et al.* [15] have listed criteria on which to base the design of health promotion information/leaflets to facilitate understanding, with a focus on both design and content. According to information processing theory [16], comprehension is central to message acceptance and is particularly important for complex health information [17]. Patient information leaflets must therefore be designed in such a way that makes them easily understood. Information about the causes and consequences of a disease must also be understood in order to motivate preventive action [18]. Images can be used to support text in order to increase health literacy, through attention, comprehension and recall of the often complex health information found in patient information leaflets [15].

Health promotion is central to the management of psoriasis, a long-term inflammatory skin condition associated with a number of problematic health behaviours. People with psoriasis are more likely than the general population to engage in excess alcohol use and smoking [19], and be overweight and sedentary [20]. These behaviours are linked to poorer psoriasis outcomes and increase the risk for cardiovascular disease (CVD) and Type 2 diabetes. Recent UK National Institute for Health and Care Excellence (NICE) guidelines for the assessment and management of psoriasis emphasise the importance of providing ‘healthy lifestyle information and support for behavioural change tailored to meet the needs of the individual’ [21]. The benefit of supported lifestyle behaviour change for other long-term conditions such as CVD [22] and diabetes [23] is well documented. However recent studies suggest that while healthcare professionals are aware of the importance of health promotion in people with psoriasis, many often miss opportunities to address such issues in consultations with psoriasis patients [24]. Furthermore, in a systematic review of adherence in people with psoriasis none of the studies addressed adherence to recommendations for healthy lifestyle [25].

Providing patients with information about healthy lifestyle in the patient waiting area can prime patients before a consultation with a healthcare professional, and may increase the likelihood of a conversation about healthy lifestyle [26, 27]. Given the evidence that: (1) the environment is important in guiding behavioural choices, and (2) health promotion and healthy lifestyle is central to psoriasis management, we conducted an observation study to investigate: the amount of information about healthy lifestyle available to patients; the sources of information (*e.g.* poster *vs.* leaflet); and the quality of information made available to patients with psoriasis in primary and secondary care health centre patient waiting areas.

## Methods

### Design

This was a non-participant observation study. Ethical approval was obtained from the University of Manchester research ethics committee (reference number: 12017). An observation check-list was designed to map the content and quality of patient leaflets and posters signposting healthy lifestyle against the ‘quality indicators’ (see section below) available in primary and secondary care health centre patient waiting areas across Northwest England. Health centres were randomly selected from a full list that was publicly available via an online database of health centres. A structured observation approach was used, whereby explicitly defined rules are followed for observing and recording a particular event/occurrence [28]. By using this approach the observer was able to record and observe the environment directly and had first-hand experience of the social phenomena under investigation [29].

### Materials

The observation check-list, developed to identify information relevant to problematic health behaviours (smoking, alcohol, weight gain, restricted activity) was refined and modified iteratively by four members of the study team. It was divided into 3 sections. Information about healthy lifestyle was also categorised based on whether it was *generic* (not specifically for patients with psoriasis; such as an information leaflet about weight loss), or *psoriasis-specific* (patient information specifically tailored to patients with psoriasis).

1. Lifestyle information available in patient waiting areas (generic versus psoriasis-specific information),
2. Lifestyle information used by practitioners to give directly to patients (generic versus psoriasis-specific); and
3. Posters/displays of available support for lifestyle.

### Data collection: structured observations

Researchers visited health centre patient waiting areas and, with permission, recorded evidence of lifestyle materials available to patients. Both primary and secondary care health centres were included in the sample.

As a secondary aim, researchers also attempted to identify the extent to which lifestyle materials were used by clinicians and given directly to patients. This was done by opportunistically asking a member of the practice team (practice manager, nurse or GP) when available, whether this was done as part of routine practice.

### Data analysis

Data were analysed using principles of content analysis [30]. Frequency rates were calculated in order to identify the number of different types of materials observed both within and between health centre waiting areas. The unit of analysis was the occurrence of lifestyle information/signposting, whether in the form of a patient information leaflet or a poster. Items were recorded if the information made reference to any problematic health behaviours (smoking, alcohol, weight gain, restricted activity).

### Quality indicators

In the absence of a suitable tool for evaluating the presence and quality of health promotion materials, we devised a quality checklist of desirable criteria to meet recommendations in relation to the following principles: a) whether health promotion information was clearly presented and of good visual condition; and b) whether health promotion displays (notice boards; carousels) were physically accessible to patients (see Table 1). The criteria were discussed and agreed upon within the study team of experienced researchers from the fields of health psychology and applied health services research based upon quality criteria identified from the literature with a focus on the design of health promotion information/leaflets [14]. All criteria used a binary scale (0 and 1).

**Table 1:** Criteria for assessment of visual condition (V) and visibility/accessibility (A) of healthy lifestyle information

•	Large, well-organised notice boards (V).
•	Large posters, with appropriately sized text which is clearly visible (V).
•	Lifestyle information is clearly visible and not obscured by other notices (*e.g.* contact details on self-referral posters for smoking cessation services are clear) (V).
•	Visually high quality information (*e.g.* no torn or crumpled leaflets) (V).
•	All information is up-to-date (*e.g.* details of exercise classes or organised walking groups previously held in the local area) (V).
•	Information is visible in the health centre waiting area or not easily accessible from the immediate waiting area (V).
•	Notice boards/displays/leaflet stands unobstructed by chairs or tables (A).
•	Notice boards/displays/leaflet stands in sight of people in waiting area (as opposed to in the corridor or outside the main waiting area) (A).

#### Visual condition of lifestyle information

Each waiting area was coded based on the observation rating and assigned to one of three levels: Level 3 (good signposting) if the recommendations were fully met, Level 2 (poor signposting) if meeting 4–5 of the criteria, and Level 1 (very poor signposting) if meeting 3 or fewer recommendations.

#### Visibility/accessibility of notice boards

Health promotion information displays (such as notice boards and leaflet stands) were also assessed on the basis of whether information was clearly visible and accessible to patients. Waiting areas were assessed on the basis of the criteria presented in Table 1. Information displays within the waiting areas were coded as having good visibility if they met all of the desirable criteria to follow recommendations relating to visibility/accessibility of information. For displays not meeting these criteria, they were coded as having poor visibility/accessibility.

#### Patient information leaflets: Layout and typographic features and use of graphics

In order to further analyse the quality of the patient information leaflets identified in the present study, a framework was used to analyse the content of a sub-sample of the lifestyle patient materials following the theoretical framework/recommendations outlined by Hartley [14] (see Table 2). To analyse the use of images in accompanying complex health information, the framework recommended by Houts *et al.* [15] was applied to the sub-sample of patient information leaflets (Table 2).

**Table 2:** Recommendations for layout and typography (summarised from Hartley [14]) and the use of images (summarised from Houts et al. [15]) in patient information leaflets

Feature	Description/Application
Margin spacing	The top, bottom and outer margins should be at least 10 mm, where inner-right and inner-left margins should be at least 25 mm.
Column format	Consistent number of columns per page should be used. Double or multiple column formats used for landscape designs. Varying column formats may confuse the reader.
Consistent spacing	Systematic spacing should be used (such as one line separating a heading from the main text or two lines separating page titles from sub-headings). Horizontal spacing (unjustified text) is also recommended.
Appropriate font size	A font size of at least 10, 12, or 14 pt is recommended for the main text, and 14, 18, or 24 for headings. Line spacing of 1.5 lines is also recommended.
Capital letters	Paragraphs of text in capital letters are hard to read, and capitals should be limited in headings.
Italicized text	Continuous italicized text is hard to read and should be limited to signalling important words or points.
Bold text	Bold text loses its effect when over-used, so should be used sparingly.
Bullet points	Should be used appropriately in outlining a series of points within a paragraph
Use of graphics to support key points	Pictures are linked with text frequently
Use of simple graphics	Simple graphics are used to understand the intended message which prevents the reader from being distracted by irrelevant details. Minimize the use of abstract symbols. When using a sequence of images explain the connection between them in simple terms
Simplified language accompanying graphics	Appropriate text should be used to accompany the images to avoid ambiguity. Language should be clear.
Closely link graphics and text	Link images and text through close proximity. Captions to describe images where possible should be written at a low literacy level, thereby aiding people with limited reading skills understand any images presented to them
Graphics should be culturally sensitive	Consider the culture of the target audience, which may affect whether people attend to the education materials. Particularly for audiences who may not have been exposed to western medicine
Involve healthcare professionals in designing the graphics	Health professionals should design the images or be involved in guiding the design of the images. This is done to successfully communicate complex information through images.
Evaluate the effects of graphics	Systematically evaluate the effects of graphics through follow interviews which can be implemented to assess: attention, understanding, remembering and adherence

## Results

Twenty-four health centres across Northwest England were observed in primary care (*n* = 21) and secondary care (*n* = 3). Characteristics of the sample are presented in Table 3.

**Table 3:** Health centre characteristics

Characteristic	Number (%) unless otherwise stated
Type of primary care centre	
GP surgery	17 (70.8)
Intermediate community dermatology clinic	4 (16.7)
Total primary care centres	21
Type of secondary care^a^	
Dermatology clinic	2 (8.3)
Hospital-based dermatology unit	1 (4.2)
Total secondary care centres	3
Patient list size	
<3000	0
3,000–5,000	4
5,000–7,000	3
7,000–9,000	2
9,000–11,000	3
11,000–13,000	3
13,000–15,000	0
15,000–17,000	1
Number of General Practitioners (range; median)	1 - 10 (5)
Number of Practice Nurses (range; median)	1 - 6 (2)
Rank of health deprivation^b^ (range; median)	4 - 20,557 (5,353)

^a^‘Hospital-based Dermatology Units’ are affiliated with hospitals and cover the full range of in-patient treatment options, where ‘Dermatology Clinics’ are usually out-patient based, may be independently run clinics or are clinics based in community settings

^b^According to the Office of National Statistics Health Deprivation index - by postcode, score out of 32782 (source: www.neighbourhood.statistics.gov.uk/dissemination/). All 32,482 neighbourhoods in England are given a health deprivation score, where the most deprived has a rank of one. Considers premature death and impairment of quality of life by poor health, and considers both physical and mental health. Measurement of morbidity, disability and premature mortality are all considered

A total of 262 sources of lifestyle information were recorded (median = 10, range = 0–40 per health setting), which are presented according to each health behaviour in Table 4.

**Table 4:** Type and number of lifestyle materials observed in primary and secondary care health centre patient waiting areas (N = 24)

Type of information	Number (%)
Generic lifestyle written information	
Smoking cessation	31 (31.6)
Alcohol reduction	16 (16.3)
Weight Loss/Diet	28 (28.6)
Exercise	22 (22.5)
Substance misuse	1 (1)
Total	98/262 (37.4)
Psoriasis-specific written information	
Smoking cessation	0 (0)
Alcohol reduction	0 (0)
Weight Loss/Diet	0 (0)
Exercise	0 (0)
Substance misuse	0 (0)
Total	0/262 (0)
Generic lifestyle information supplied to patients by practitioners^a^	
Smoking cessation	11 (25.6)
Alcohol reduction	6 (14)
Weight Loss/Diet	16 (37.2)
Exercise	9 (20.9)
Substance misuse	1 (2.3)
Total	43/262 (16.4)
Psoriasis-specific lifestyle information supplied to patients by practitioners^a^	
Smoking cessation	2 (25)^b^
Alcohol reduction	2 (25)^b^
Weight Loss/Diet	2 (25)^b^
Exercise	2 (25)^b^
Total	8/262 (3.1)
Posters/displays of available support for lifestyle	
Smoking cessation	21 (18.6)
Alcohol reduction	14 (12.3)
Weight Loss/Diet	22 (19.5)
Exercise	56 (49.6)
Total	113/262 (43.1)
Total number of materials observed	262

^a^Analysis based on data collected from 12 health centres. Data were unable to be collected from the remaining health centres (*n* = 12)

^b^Relates to one patient information leaflet recorded in two different practices. Leaflet contains information applicable to all health behaviours

The majority of lifestyle signposting occurred through the use of posters (*n* = 113; 43.1 %) and generic lifestyle materials (leaflets and flyers) specifically for patients to take away (*n* = 98; 37.4 %). Of the 24 health centres, themed notice boards were present in 10 (41.7 %), of which five (20.8 %) were generic lifestyle notice boards, one (4.2 %) was lifestyle-related in the context of diabetes self-management, one (4.2 %) was a general dermatology notice board, and three (12.5 %) were psoriasis-specific notice boards. The remaining health centres (*n* = 14; 58.3 %) did not contain themed notice boards.

Of the lifestyle information offered directly to patients (51 examples), collected from 12 health centres, 84.3 % (*n* = 43) were categorised as generic lifestyle, whilst 15.7 % (*n* = 8) were categorised as psoriasis-specific or tailored. Psoriasis-specific lifestyle written information was not freely available to patients in waiting areas of either primary care or secondary care health centres.

### Breakdown by *health behaviour*

There was considerable variation in the type of lifestyle signposting for the different health behaviours. Generic written information was more likely to be related to smoking cessation (*n* = 31; 31.6 %) and weight loss/diet (*n* = 28; 28.6 %) than other health behaviours. Lifestyle information used by clinicians to give to patients was mainly focused on weight loss/diet (*n* = 16; 37.2 %). Only one source of psoriasis specific written information was identified (a psoriasis leaflet advising of CVD risks related to unhealthy behaviours) and this was present in two health settings. Posters/displays signposting lifestyle support predominantly concerned weight loss (*n* = 56; 49.6 %). (See details in Table 4).

### Breakdown by *setting* (primary care *vs.* secondary care)

Signposting for lifestyle was more visible within primary care health centres (median = 10, range 0–40) than secondary care health centres (median = 11, range 0–15) and this was almost exclusively generic information about healthy lifestyle. A small number of lifestyle psoriasis-specific practitioner materials were recorded in both primary care (*n* = 4) and secondary care (*n* = 4).

### Breakdown by *type of service* (specialist psoriasis/dermatology health centre *vs.* general practice health centre)

Signposting for lifestyle was more visible general practice health centres (median = 10, range 0–40) than specialist psoriasis/dermatology health centres (median = 3, range 0–29) and this was almost exclusively generic information about lifestyle, with the exception of a limited number of psoriasis-specific practitioner materials in the specialist services settings (*n* = 8). (See Table 5).

**Table 5:** Type and number of lifestyle materials observed in specialist Psoriasis/Dermatology centres (N = 7) compared with general practice health centres (N = 17)

Type of information	Number (%) in Specialist Psoriasis/Dermatology Health Centres	Number (%) in Primary care Health Centres
Generic lifestyle written information		
Smoking cessation	10 (37)	21 (29.6)
Alcohol reduction	2 (7.4)	14 (19.7)
Weight Loss/Diet	8 (29.6)	20 (28.2)
Exercise	7 (25.9)	15 (21.1)
Substance misuse	0 (0)	1 (1.4)
Total	27/262 (10.3)	71/262 (27.1)
Psoriasis-specific written information		
Smoking cessation	0 (0)	0 (0)
Alcohol reduction	0 (0)	0 (0)
Weight Loss/Diet	0 (0)	0 (0)
Exercise	0 (0)	0 (0)
Substance misuse	0 (0)	0 (0)
Total	0/262 (0)	0/262 (0)
Generic lifestyle information supplied to patients by practitioners^a^		
Smoking cessation	4 (26.7)	7 (25)
Alcohol reduction	1 (6.7)	5 (17.9)
Weight Loss/Diet	6 (40)	10 (35.7)
Exercise	4 (26.7)	5 (17.9)
Substance misuse	0 (0)	1 (3.6)
Total	15/262 (5.7)	28/262 (10.7)
Psoriasis-specific lifestyle information supplied to patients by practitioners^a^		
Smoking cessation	2 (25)^b^	0
Alcohol reduction	2 (25)^b^	0
Weight Loss/Diet	2 (25)^b^	0
Exercise	2 (25)^b^	0
Total	8/262 (3.1)	0/262 (0)
Posters/displays of available support for lifestyle		
Smoking cessation	5 (55.5)	16 (15.4)
Alcohol reduction	0 (0)	14 (13.5)
Weight Loss/Diet	1 (11.1)	21 (20.2)
Exercise	3 (33.3)	53 (51)
Total	9 (3.4)	104/262 (40)
Total number of materials observed	59	203

^a^Analysis based on data collected from 12 health centres. Data were unable to be collected from the remaining health centres (*n* = 12)

^b^Relates to one patient information leaflet recorded in two different practices. Leaflet contains information applicable to all health behaviours

### Quality indicators

Results of the quality assessment of the lifestyle materials identified are presented according to each indicator.

#### Visual condition of lifestyle information

**Fig. 1:**
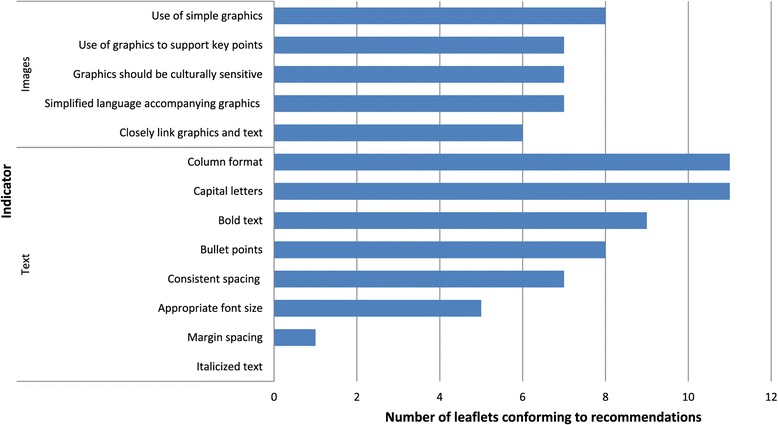
Analysis of layout and typography and the use of images in a sub-sample of randomly selected patient information leaflets (*n* = 13)

Each waiting area was coded based on the observation rating (criteria listed in Table 1). Of the health centres included in the study, two (11.8 %) were coded as Level 3 signposting (good signposting), eleven (64.7 %) were coded as Level 2 signposting (poor signposting), and one (5.9 %) was coded as Level 1 signposting (very poor signposting). Three (17.6 %) health centres contained no lifestyle information. The remaining health centres (*n* = 7) were not assessed at the request of the health centre. Examples of poor lifestyle information are presented in Fig. 1 for illustrative purposes.

#### Visibility/accessibility of notice boards

Notice boards were clearly visible and accessible in eight (36.4 %) health settings observed. Fourteen (63.6 %) were assessed as having notice boards that were not easily accessible/visible. The remainder (*n* = 2) were not assessed at the request of the health centre.

Analysis of patient information leaflets was based on a sub-sample of the leaflets identified in the study (*n* = 13) and were randomly sampled across 11 of the health centres.

### Layout and typographic and use of graphics/images in patients information leaflets

**Fig. 2:**
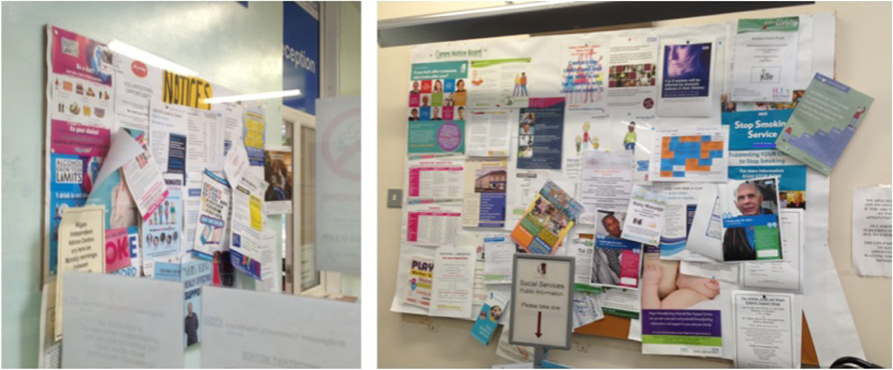
Examples of low quality healthy lifestyle signposting

Results of the layout and typographical features and the use of graphics/images analysis are presented in Fig. 2. These patient information leaflets conformed to guidelines in relation to column format (*n* = 11; 84.6 %) and the use of capital letters (*n* = 11; 84.6 %) but low agreement with guidelines related to the use of bold text (*n* = 9; 69.2 %), bullet points (*n* = 8; 61.5 %), use of consistent spacing (*n* = 7; 53.8 %), and appropriate font size (*n* = 5; 38.5 %). There was little agreement with margin spacing guidelines (*n* = 1; 7.7 %) and no agreement with use of italicized text.

Of the indicators used to analyse the use of graphics/images in patient information leaflets, strongest agreement was shown with the use of simplified graphics (*n* = 8; 61.5 %). Leaflets showed modest agreement with guidelines related to the use of graphics to support key points (*n* = 7; 53.8 %), cultural sensitivity of graphics (*n* = 7; 53.8 %), and the use of simplified language to support graphics (*n* = 7; 53.8 %), and proximity of graphics and text (*n* = 6; 46.2 %). It was not possible to discern whether health professionals were involved in the design of the leaflets, and whether the use of graphics was evaluated with patient groups. Therefore these guidelines were not included in the final analysis.

## Discussion

This is the first study to examine the presence and quality of health promotion information available to patients with psoriasis in primary and secondary care waiting areas. There are three important findings: (1) there was little attempt to promote healthy lifestyle as an important aspect of psoriasis management in the clinic environment; (2) generic patient materials were of poor quality and were poorly displayed; and (3) healthy lifestyle information failed to conform to evidence-based health promotion strategies, such as principles related to choice architecture. In order to conform to such principles information must be placed in the environment in a clear, visible and accessible way.

Our study demonstrates there is little attempt to provide tailored psoriasis-specific patient information about health promotion. Variation between health care centres in terms of the number and type of generic patient materials suggests a lack of patient exposure to information about healthy lifestyle in general. Information often appeared disorganised and cluttered, and leaflets were often very poor in terms of visual appearance (in quality and how they were displayed on noticed boards).

It is possible that improving the amount and quality of generic information would serve to increase patient understanding of the benefits of a healthy lifestyle. However, given the role of behavioural factors in the maintenance and exacerbation of psoriasis (such as skin flare-ups), as well as associated CVD risk, psoriasis-specific information about behaviour change should be an important aspect of psoriasis management. Future studies should aim to examine the effectiveness and feasibility of providing psoriasis-specific information versus generic information for increasing healthy lifestyles.

Health centre waiting areas are a prime location for promoting healthy lifestyles where people have time to read and assimilate information. Restructuring the environment to make health promotion materials more accessible and understandable could be a way of engaging patients in behavioural change. Health promotion messages when embedded in people’s physical environment can prompt behavioural change [31]. Interestingly, Kerr *et al.* found that only posters larger than A3 were effective for health promotion in the context of an immediate behavioural choice, suggesting studies such as this can be used to inform recommendations for the size of more effective materials in the context of the health centre environment. Posters in the current study generally did not conform to these guidelines. Patient information leaflets are one of the most commonly used methods of communicating health messages to patients [32]. Thus, the need to develop health information which is easily understood and then acted upon by the general public is crucial [33].

Whilst research around choice architecture (shaping the environment to encourage healthier behavioural choices) has been conducted in a range of settings to encourage positive behaviour change, this knowledge has yet to be applied to the immediate healthcare environment. Our findings demonstrate missed opportunities to promote behaviour change. The simple addition of clearer information about healthy lifestyle in settings where patients with psoriasis are managed could be an inexpensive way of ‘priming’ people for change. Priming patients before a consultation with their healthcare professional may increase the likelihood of discussions about behaviour change. For example receiving information about behaviour change in the waiting room via patient information leaflets or posters, preceding the healthcare professional-patient interaction may facilitate behaviour change [26, 27].

### Strengths and limitations of the study

Health promotion signposting for people with psoriasis was examined systematically using key criteria from the literature and drawing on the expertise within the study team. The observational approach to this study meant that the researchers were able to gain first-hand experience of the healthcare settings in which the study took place, specifically from the patient’s perspective.

There are several limitations to this work. Firstly, due to time and logistical restrictions, only one observer was able to carry out the coding at any one time, thus we were unable to calculate inter-rater reliability. However we attempted to overcome this by using a pre-determined, clearly specified observation check-list comprising well-defined categories to reduce any misinterpretation or likelihood of bias [28]. Secondly, due to the cross-sectional nature of the study we were unable to assess whether the display of health promotion material changed across different time points. However we attempted to overcome this by obtaining a varied sample of health centres across a large geographical area which attempted to capture the widest possible variation in health promotion displays. Thirdly, with regard to opportunistically asking practitioners whether they distributed information leaflets to patients, whilst we acknowledge this may have the potential for selection bias, we were still able to collect data from half of the health centres involved in the study. Finally, we present a sub-sample of the total number of participant information leaflets analysed according to an existing framework for text and imagery recommendations in participant information. These were randomly selected, and having found no examples of high quality materials, we are confident this is a fair representation of the sample. However future studies should aim to conduct a more in-depth examination of information leaflets, such as examining theoretical content, or the type of advice given (*e.g.* practical versus generic advice).

### Recommendations for practice

Evidence from the field of health psychology and health literacy could be used to inform the development of: (1) high quality patient materials; and (2) effective lifestyle signposting. This is particularly the case for psoriasis-related patient materials. Further research should aim to use evidence based approaches to design, layout and display behaviour change information. The practical application of evidence based approaches in the development of patient information leaflets to guide communication of health information is beginning to be recognised [34, 35]. It is therefore necessary that improvements to lifestyle signposting must consider the empirical evidence to develop and implement theory-driven interventions.

There is strong empirical evidence that theory-based interventions can be used successfully to target key behavioural constructs in shaping behaviour [36], such as improving self-efficacy, attitudes, and beliefs [37, 38]. Restructuring the environment is also recognised as a key component of interventions designed to change behaviour [39]. Further research should explore how the environment can be used to promote healthy lifestyle beyond effective patient information displays. Whilst brief effects on behavioural change have been observed, further work is needed to establish whether such effects can be maintained [40].

## Conclusions

Our study identified an urgent need to provide effective healthy lifestyle signposting for patients with psoriasis, consistent with the NICE guideline on management of psoriasis. Current practice is not utilising evidence-based approaches to the design and presentation of health promotion information about healthy lifestyle in the clinical environment and opportunities are being missed to aid behaviour change.

## Abbreviations

Lifestyle: Lifestyle behaviour change
CVD: Cardiovascular disease
NICE: National institute for health and care excellence
